# Hepatic metabolomic profiling changes along with postnatal liver maturation in breeder roosters

**DOI:** 10.1242/bio.028944

**Published:** 2018-01-15

**Authors:** Shengru Wu, Yanli Liu, Liqin Zhu, Di Han, Musa Bello Bodinga, Xiaojun Yang

**Affiliations:** College of Animal Science and Technology, Northwest A&F University, Yangling, Shaanxi 712100, P. R. of China

**Keywords:** Brooding period, Chicken, Growing period, Metabolomics, Postnatal liver metergasis

## Abstract

To understand the hepatic metabolic changes during postnatal liver maturation process in breeder roosters, we investigated the hepatic metabolites composition of 1-day-old, 42-day-old, and 35-week-old breeder roosters using gas chromatography-mass spectrometer (GC-MS). Comprehensive multivariate data analyses were applied to identify the distinguishing metabolites of liver. 84 different kinds of distinguishing metabolites were identified between the livers of 1-day-old and 42-day-old breeder roosters, and 58 different kinds of distinguishing metabolites were identified between the livers from 42-day-old and 35-week-old breeder roosters. Further pathway annotations revealed that the hepatic metabolism was extensively remodeled during the postnatal liver maturation process. The antioxidant capacity of the liver and metabolism of carbohydrates, proteins, amino acids, fats, cholesterols, nucleic acids, and vitamins were all significantly changed at different growing periods after birth. Specifically, we found that the hepatic amino acid metabolic function was continuously enhanced from 1-day-old to 35-week-old roosters. However, the glucose and lipid metabolic functions were weakened from 1-day-old to 42-day-old roosters and then elevated from 42-day-old to 35-week-old roosters. In conclusion, the present study revealed that the metabolomic changes are related to the adaption of liver functions in breeder roosters.

## Abstract (precis)

**Summary:** This study revealed that hepatic metabolomic changes are related to functional adaption in breeder roosters, which contribute to a better understanding of the hepatic metabolites composition differences during different periods.

## INTRODUCTION

Liver, being the most important organ of metabolic system after birth, plays major roles in nutrient homeostasis including the synthesis, metabolism, and transport of carbohydrates, proteins, and fats. However, liver is mainly hematopoietic in the embryo (Oliver et al., 1983; Perry et al., 1983; Shelly et al., 1989) and then converted into a major metabolic tissue at adult stage, which indicates that matured hepatic metabolic function is extensively remodeled after birth (Si-Tayeb et al., 2010; Bhate et al., 2015). Recent studies in mice and pigs reveals that the metabolic functions of livers have been continuously changing during the postnatal liver maturation process using RNA sequencing (Li et al., 2010; Peng et al., 2014). Thus, we proposed that the chicken hepatic functions will also be remodeled after birth, as reported in other animal species.

One breeder rooster produces around 1000 broiler chicks per year (Berghof et al., 2013). A good hepatic metabolic condition is important to the health and reproductive ability of breeder roosters (Kalmar et al., 2013), which could further increase the usability of the breeder roosters. Meanwhile, the liver functions were continuously changed to suit their growing condition. Hence, it is important to clarify the hepatic metabolic characteristics of breeder roosters in different growing periods.

Metabolomics, defined as the analysis of metabolome using high-throughput approaches, is an emerging powerful discovery tool that can quantitatively measure a complete set of small molecular metabolites in biological samples (Sun et al., 2015). Metabolomics analysis of these metabolites can comprehensively characterize the metabolic mechanism of biological systems under internal or environmental stimulating factors (Tang and Wang, 2005). Alterations in the metabolome can provide an insight into the molecular aspects of pathogenesis of complex disease (Yue et al., 2016), dietary exposures (Shi et al., 2013) as well as organ development (Hazard et al., 2011) in different growing or physiological periods of animal (Sun et al., 2015; Dervishi et al., 2017). Herein, gas chromatography-mass spectrometer (GC-MS)-based metabolomics is a suitable approach to study the hepatic metabolic condition changes from birth to adult in breeder rooster.

In the present study, we systematically analyzed the hepatic metabolites and the alterations of hepatic metabolome in different developmental periods of breeder rooster using GC-MS metabolomics. Moreover, pathway analyses of the identified distinguishing metabolites were further carried out to clear the metabolic pathways related to postnatal liver development. These metabolomic profiles could be helpful to clarify the differences of hepatic metabolism in different periods of breeder roosters.

## RESULTS AND DISCUSSION

### Hepatic metabolic changes of breeder roosters during the brooding period

**Fig. 1.**
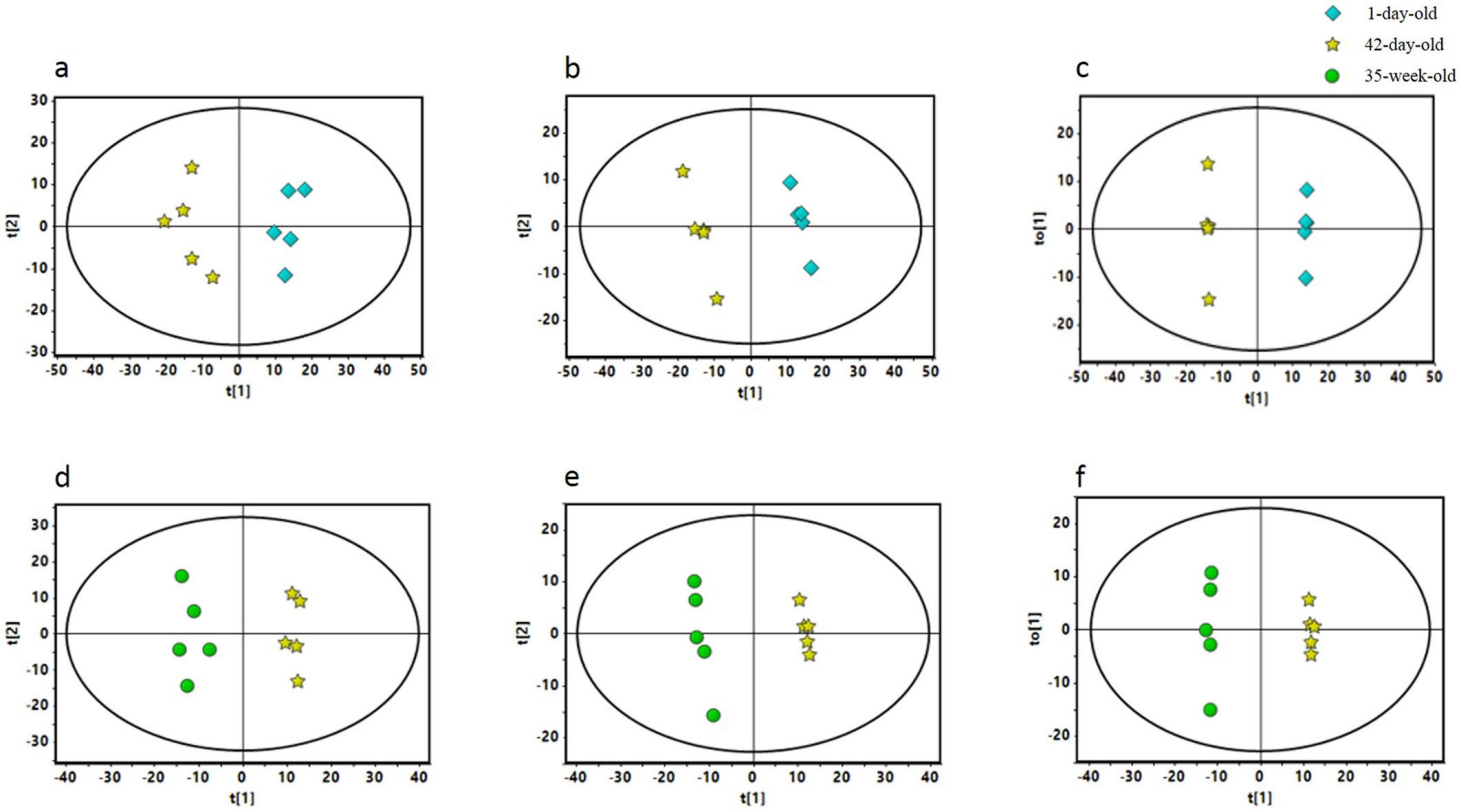
**Distribution of the liver samples from 1-day-old, 42-day-old, and 35-week-old breeder roosters.** (A) The principal component analysis (PCA), (B) the partial least squares discriminant analysis (PLS-DA), and (C) the orthogonal projection of latent structures-discriminant analysis (OPLS-DA) of liver samples of five 1-day-old and five 42-day-old breeder roosters. (D) PCA, (E) PLS-DA, and (F) OPLS-DA analyses of liver samples of five 42-day-old and five 35-week-old breeder roosters. t(1), first principal component; t(2), second principal component; to(1), orthogonal component.

In order to comprehensively understand the metabolic changes of breeder roosters during brooding period, metabolomic studies of liver tissues were performed to identify the distinguishing metabolites between 1-day-old and 42-day-old breeder roosters. Initially, we performed a principal component analysis (PCA) (R^2^X=0.545) to examine the interrelationship between different groups. The liver samples from the 1-day-old and 42-day-old breeder roosters were distributed in two separate areas, indicating a markedly different metabolome between 1-day-old and 42-day-old breeder roosters (Fig. 1A). Meanwhile, a partial least squares discriminant analysis (PLS-DA) (R^2^Y=0.997, Q^2^=0.95, Fig. 1B) was performed to maximize the difference of metabolic profiles between the two groups and allow for metabolite recognition, which demonstrated a clearer separation between the 1-day-old and 42-day-old breeder roosters for liver metabolomic profiles.

**Fig. 2.**
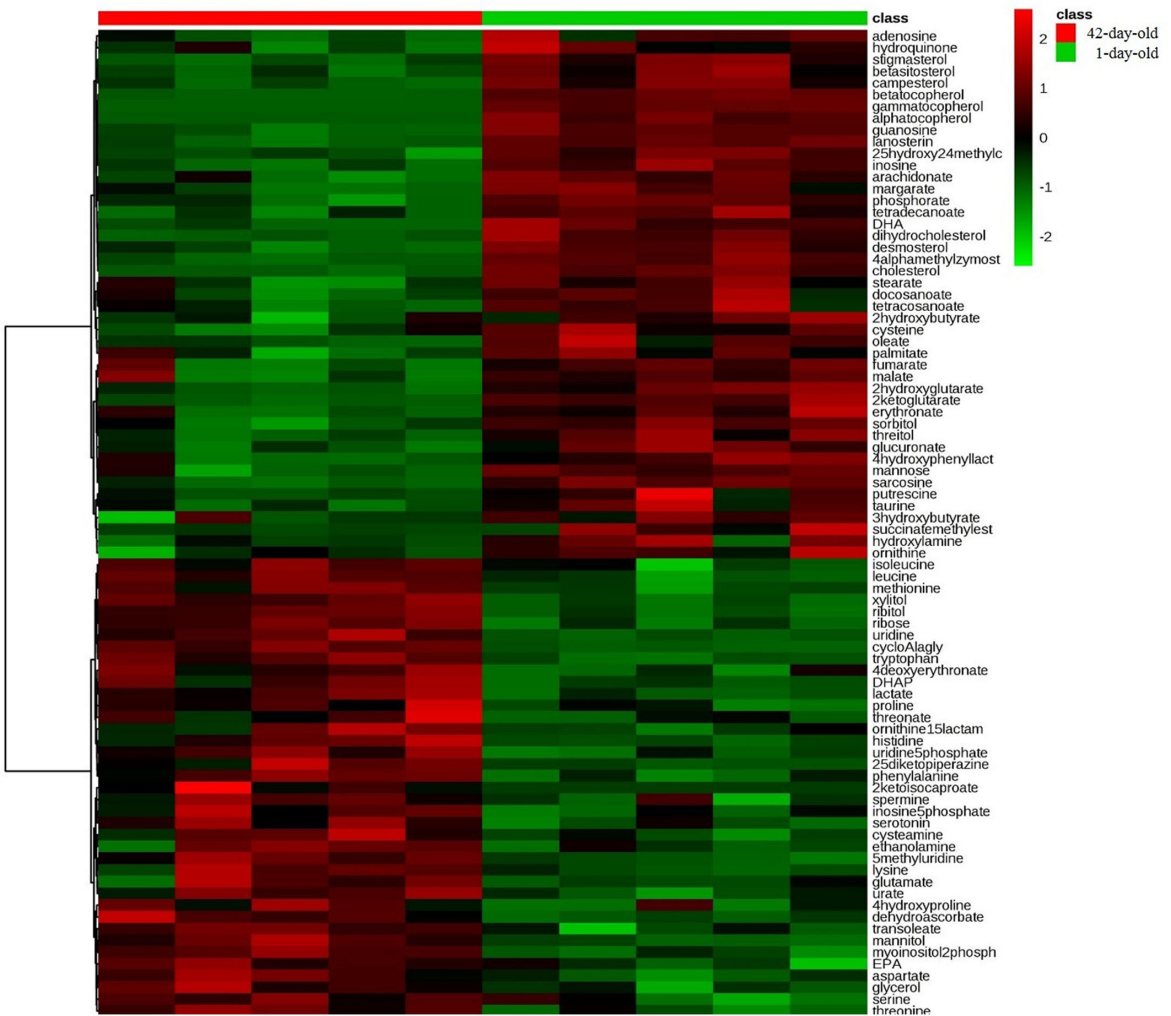
**The heat map of distinguishing metabolites in livers between the 1-day-old and 42-day-old breeder roosters.** The up-regulated metabolites are shown in red color, whereas the down-regulated metabolites are presented in green color. DHA, docosahexenoic acid; DHAP, dihydroxyacetone phosphate.

Furthermore, a supervised models orthogonal projections to latent structures discriminant analysis (OPLS-DA) (R^2^X=0.516, R^2^Y=0.997, Q^2^=0.93) was performed to identify distinguishing hepatic metabolites between the breeder roosters during the brooding period (Fig. 1C). The variable importance in the projection (VIP) value, *P* value, along with the fold change (FC) value were obtained. The VIP values of the variables reflected their importance, and the variables with highest VIP values could be the potential biomarkers. Referring to the criteria with VIP value>1 and *P* value<0.05, a total of 84 distinguishing metabolites were identified (Fig. 2). Compared with the hepatic metabolites of 1-day-old chicks, 39 up-regulated metabolites and 45 down-regulated metabolites in 42-day-old breeder roosters were identified. Specifically, the fumaric acid, 2-ketoglutaric acid, and malic acid, which are related to tricarboxylic acid (TCA) cycle (Sunny et al., 2011), were significantly decreased with postnatal liver development process during the brooding period. Meanwhile, many metabolites involved in the fatty acid and steroid metabolism process were also significantly decreased, which included 25-hydroxy-24-methylcholesterol, 4α-methylzymosterol, beta-sitosterol, campesterol, desmosterol, dihydrocholesterol, lanosterin, stigmasterol, cholesterol, arachidonic acid, docosahexaenoic acid, docosanoic acid, margaric acid, oleic acid, palmitic acid, stearic acid, tetracosanoic acid, and tetradecanoic acid (Nguyen et al., 2008; Syggelou et al., 2012). However, other metabolites associated with pentose phosphate pathway, gluconeogenesis, and glycerophospholipid metabolism process were remarkably increased, including ribose, lactic acid, dihydroxyacetone phosphate, ethanolamine, and myo-inositol-2-phosphate (Krebs and Eggleston, 1974; Jones et al., 1997). Additionally, the concentration of several amino acids and metabolites related to amino acid metabolism, such as aspartic acid, proline, glutamic acid, methionine, serine, threonine, histidine, lysine, tryptophan, isoleucine, leucine, 4-hydroxyproline, and serotonin, were all significantly increased. This indicated that these amino acids were crucial for the growth and development of breeder rooster during the brooding period (Wu, 2009). Meanwhile, the spermine, which has been proved to take part in the antioxidant process in previous study (Eisenberg et al., 2016), was significantly increased in 42-day-old chickens. This inspired us that the antioxidant function could be improved during the brooding period. These results displayed an extensive hepatic metabolic changes during brooding period of breeder roosters.

**Fig. 3.**
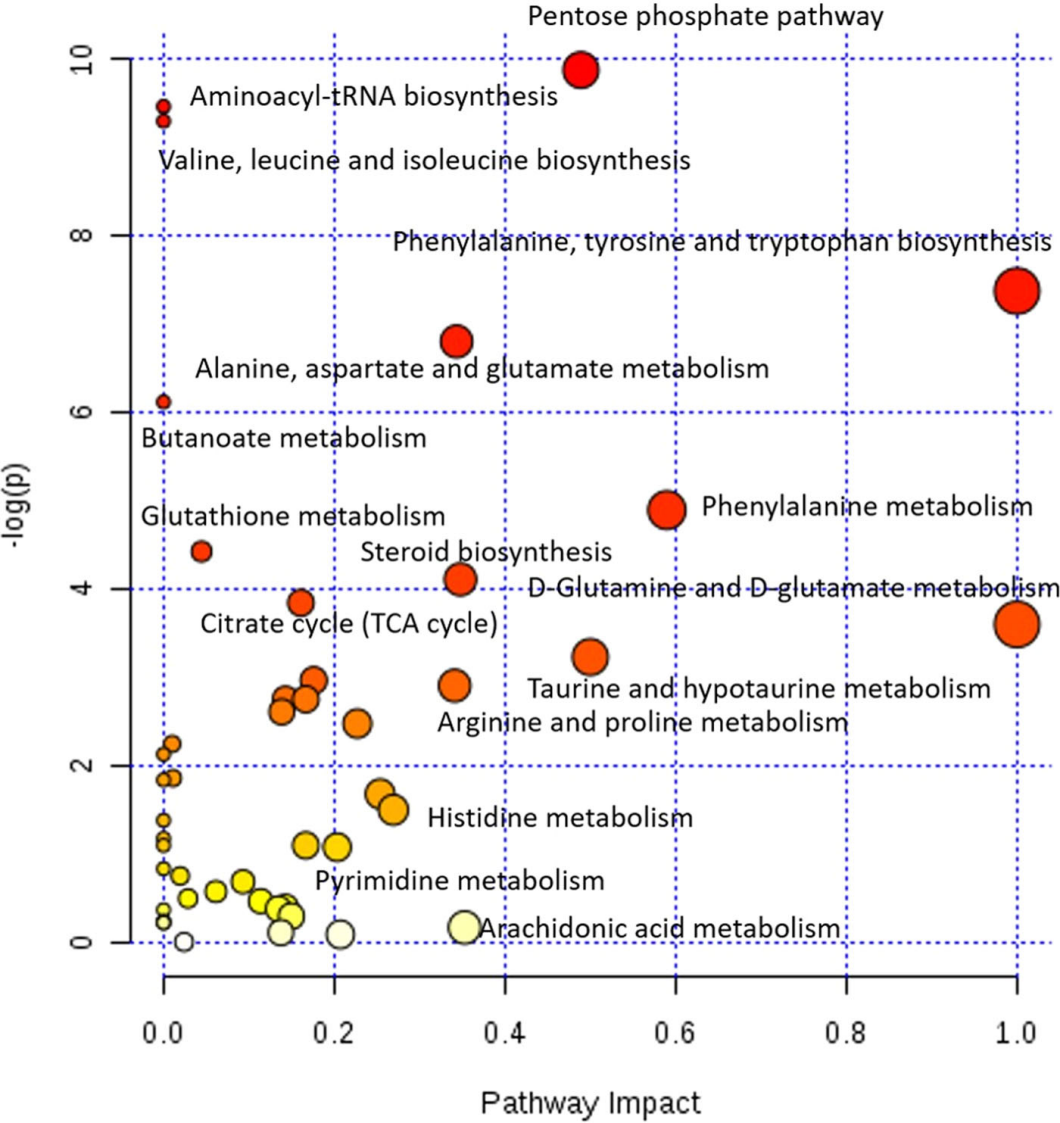
**Pathway analysis showing changes in the metabolism of breeder roosters' livers during the brooding period.**

Furthermore, metabolic pathway analysis was performed using MetaboAnalyst. The distinguishing metabolites revealed that 9 biochemical pathways were significantly altered (Table 1). By combining the topology with a powerful pathway enrichment analysis, the significantly altered pathways included the D-glutamine and D-glutamate metabolism, taurine and hypotaurine metabolism, arginine and proline metabolism, steroid biosynthesis, alanine, aspartate and glutamate metabolism, as well as amino acyl-transfer ribonucleic acid (tRNA) biosynthesis (Fig. 3). Considering the results of distinguishing metabolites and enriched pathways, the fatty acid and steroid metabolic function in 1-day-old chicks were promoted, consistent with previous reports (Janke et al., 2004; Sato et al., 2006). Moreover, the metabolism of protein and several amino acids were enhanced during the brooding period, which includes the glutamine, glutamate, arginine, proline, alanine, and aspartate. Previous studies have also proved that these 6 amino acids were crucial for the growth and development of chicken during the brooding period (Fifkova and Van Harreveld, 1970; Bartell and Batal, 2007; Suenaga et al., 2008; Balázs et al., 2012; Kralik et al., 2014). In conclusion, except for the weakening of lipid and steroid metabolic functions, the enhanced metabolic process of amino acids should be given special consideration during the brooding period

**Table 1.**
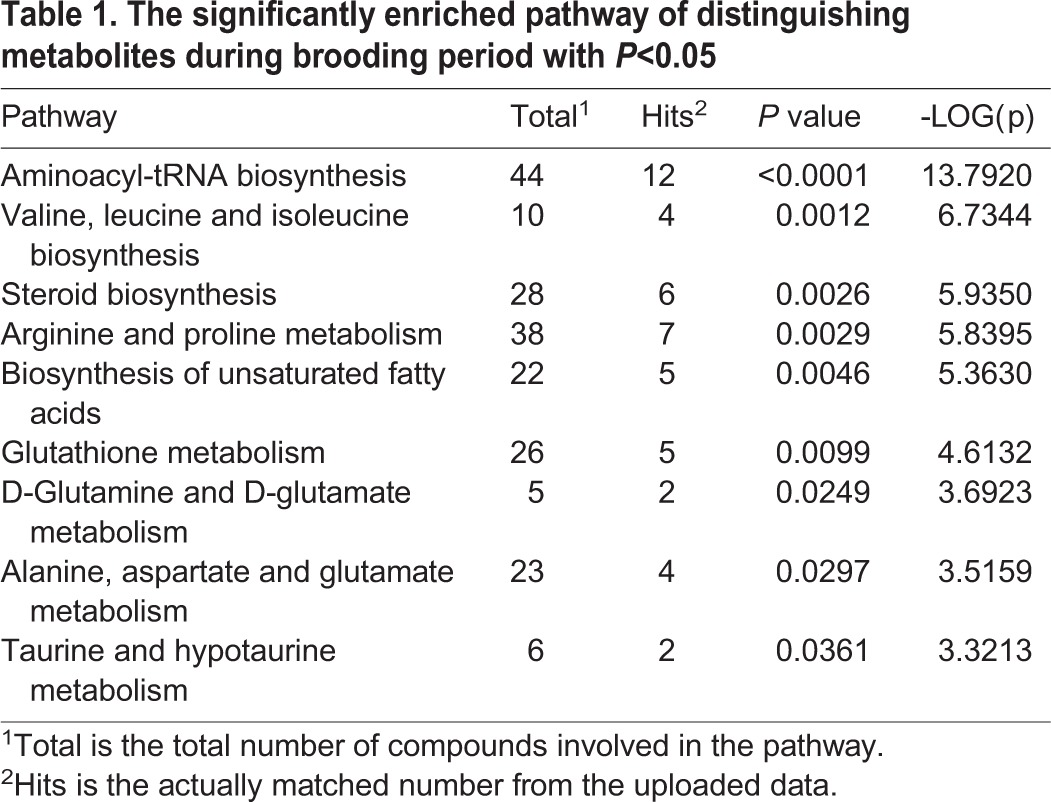
**The significantly enriched pathway of distinguishing metabolites during brooding period with *P*<0.05**

### Hepatic metabolic changes during the growing period of breeder roosters

**Fig. 4.**
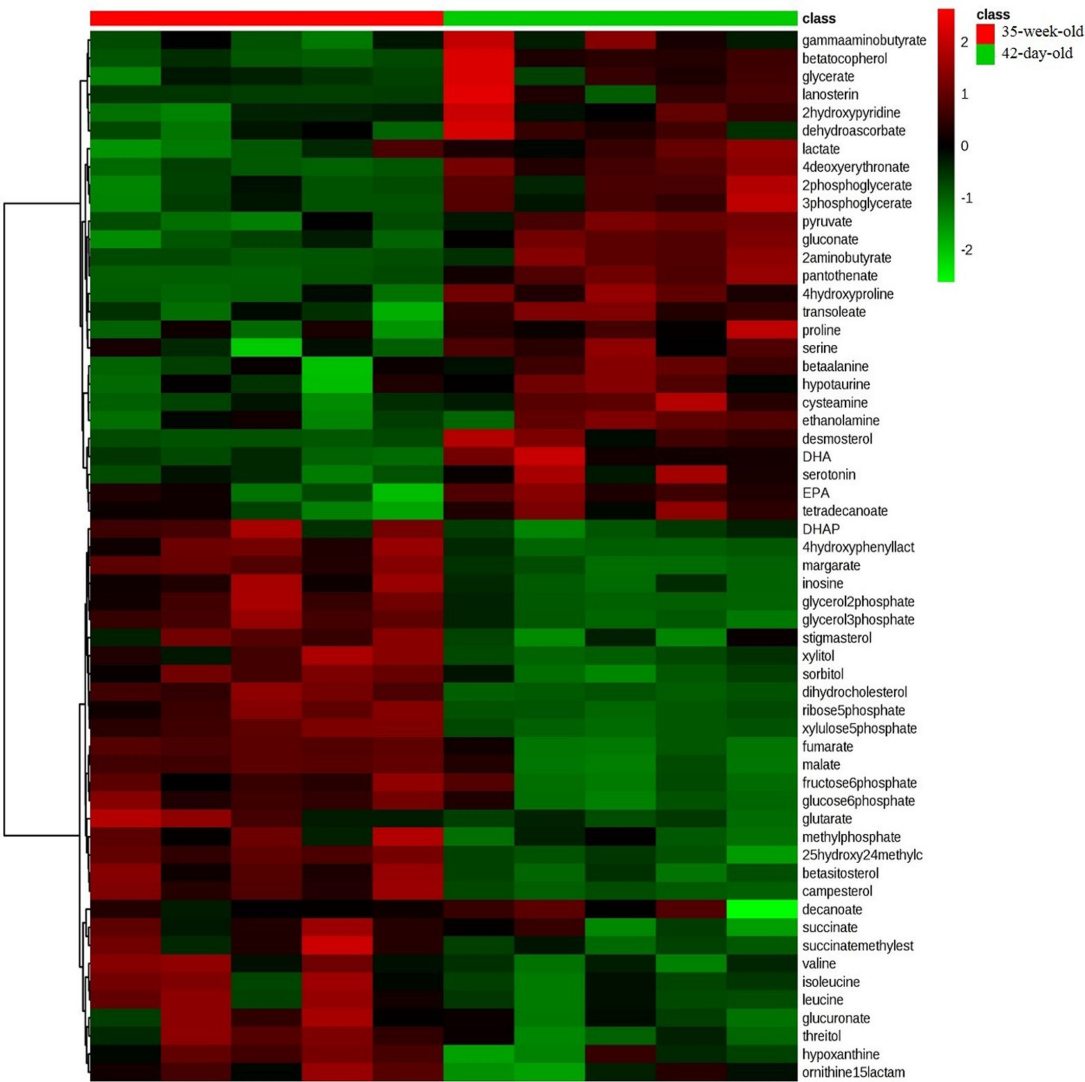
**The heat map of distinguishing metabolites in livers between the 42-day-old and 35-week-old breeder roosters.** The up-regulated metabolites are shown in red color, whereas the down-regulated metabolites are presented in green color. DHA, docosahexenoic acid; DHAP, dihydroxyacetone phosphate; EPA, eicosapentaenoic acid.

**Fig. 5.**
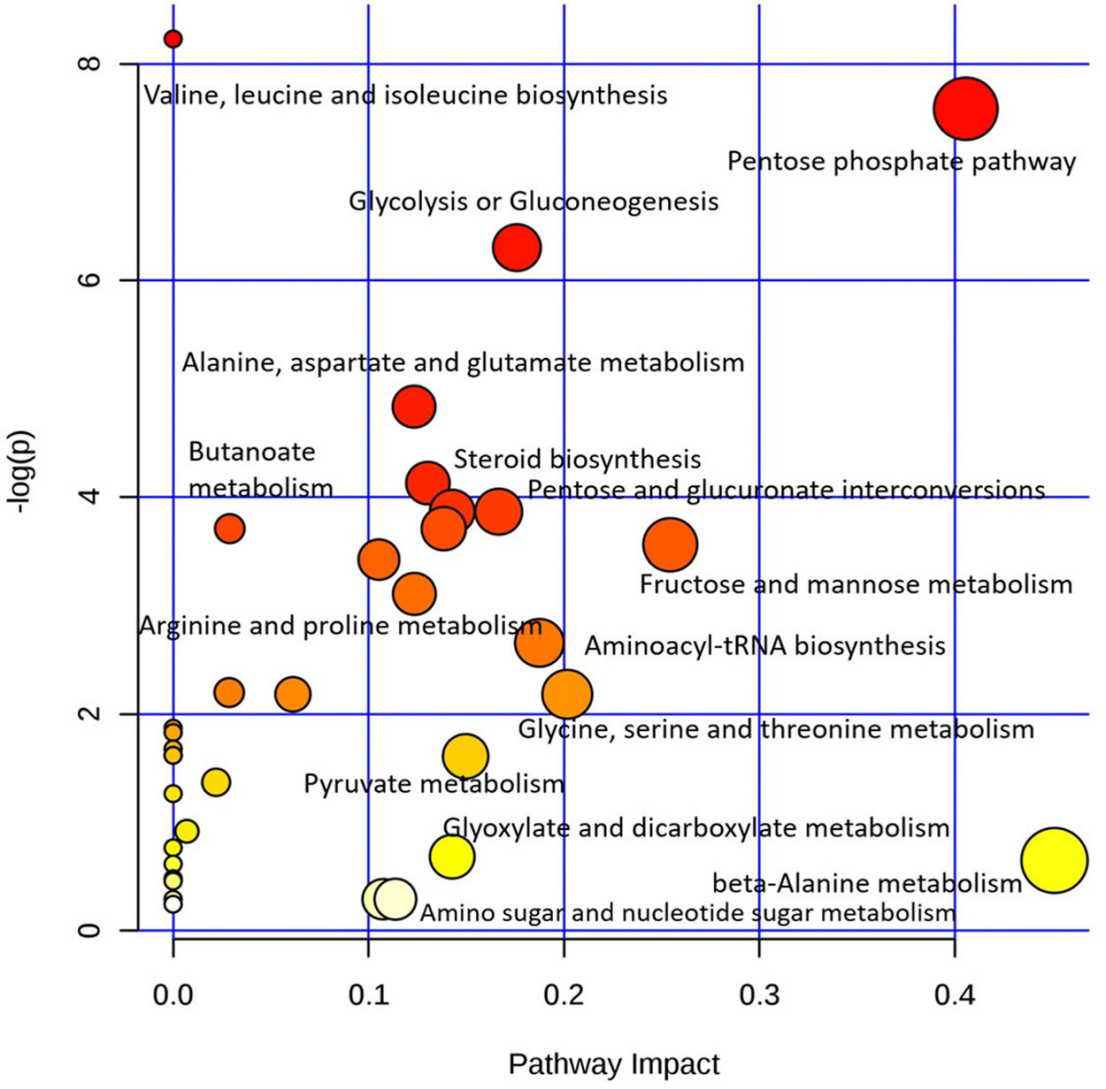
**Pathway analysis showing changes in the metabolism of breeder roosters' livers during the growing period.**

We further comprehensively studied the hepatic metabolic changes between 42-day-old and 35-week-old breeder roosters. The PCA (R^2^X=0.597, Fig. 1D) and PLS-DA score plots (R^2^Y=0.998, Q^2^=0.963, Fig. 1E) were also well distinguished from these two ages of breeder roosters, which has indicated a markedly different metabolome. The supervised OPLS-DA (R^2^Y=0.998, Q^2^=0.966, Fig. 1F) analysis was then performed and a total of 58 significantly distinguishing metabolites were screened (Fig. 4), of these, 28 metabolites were up-regulated and 30 metabolites were down-regulated in the 35-week-old breeder roosters. The involved metabolic pathways were further mapped and we found that 12 metabolic pathways involved in amino acid metabolism, lipid and steroid metabolism, as well as glycometabolism were significantly changed (Table 2, Fig. 5).

**Table 2.**
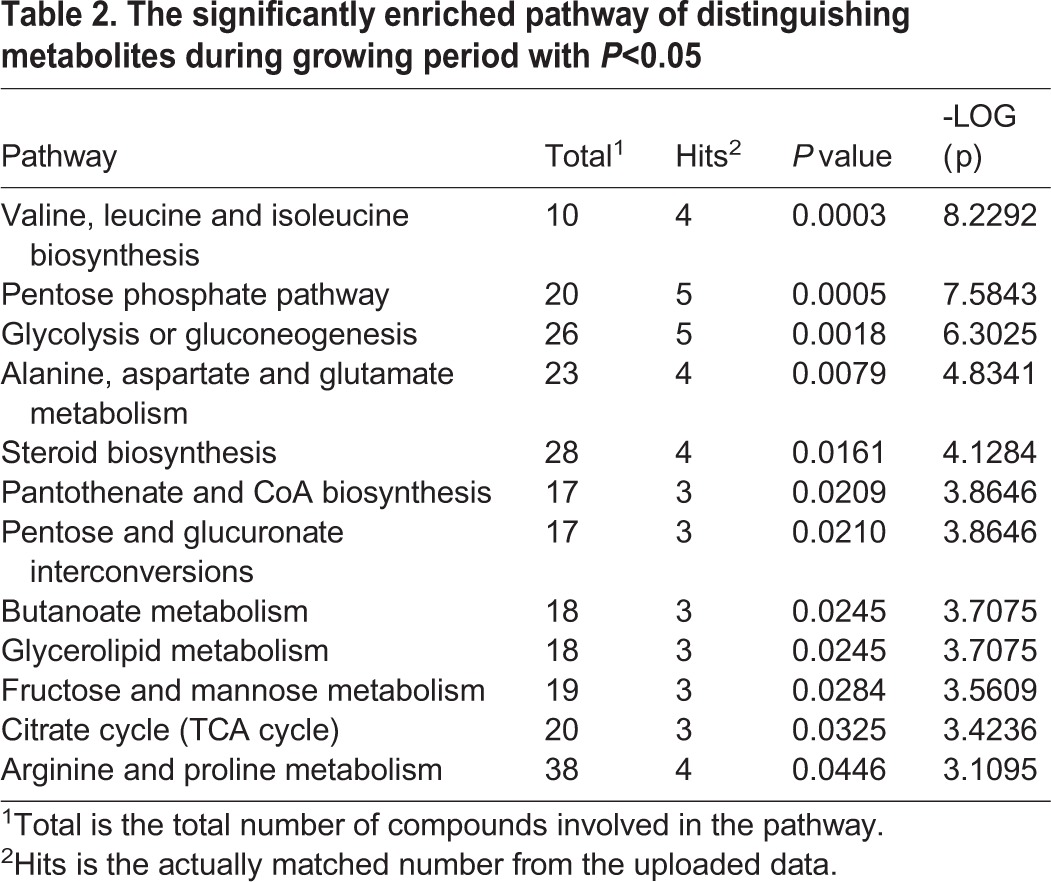
**The significantly enriched pathway of distinguishing metabolites during growing period with *P*<0.05**

Based on our results, we found that the livers of 35-week-old breeder roosters have a better metabolic efficiency in pentose phosphate pathway, TCA cycle, and glycolysis/gluconeogenesis. This could be from the increased concentration of fumaric acid, succinic acid, malic acid, glucose-6-phosphate, fructose-6-phosphate, and dihydroxyacetone phosphate, as well as the decreased concentration of 3-phosphoglyceric acid, 2-phosphoglyceric acid, and glyceric acid (Sunny et al., 2011). Similarly, the metabolic efficiency of most amino acid metabolic pathways, including biosynthesis of valine, leucine and isoleucine, as well as the metabolism of alanine, aspartate and glutamate, were significantly increased (Wu, 2009). Moreover, the metabolic efficiency related to lipid metabolic process, including lipid degradation, as well as steroid biosynthesis, were significantly increased, which could be due to the increased concentration of 25-hydroxy-24-methylcholesterol, beta-sitosterol, campesterol, dihydrocholesterol, stigmasterol, glycerol 2-phosphate, and glycerol 3-phosphate as well as the decreased concentration of decanoic acid, docosahexaenoic acid, eicosapentaenoic acid, tetradecanoic acid, and trans-oleic acid (Nguyen et al., 2008; Syggelou et al., 2012). To sum up, the improved metabolic functions will be our focus during the growing period.

### Comparison of the metabolic profiles of livers among different ages

**Fig. 6.**
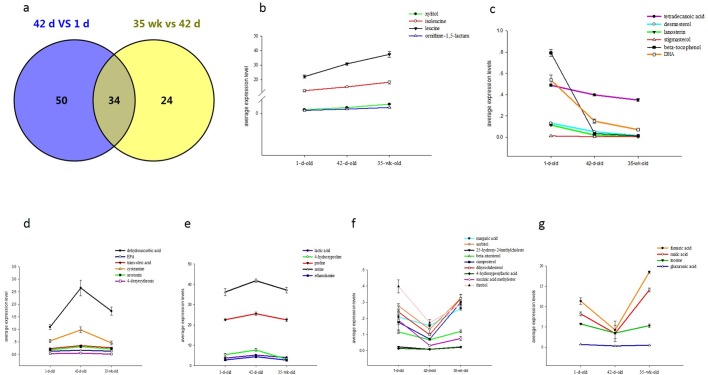
**The co-altered metabolites in both brooding and growing periods which have different variation tendency.** (A) Venn diagram analysis of the metabolic profiles of livers among different ages; (B) continually increased metabolites; (C) continually decreased metabolites throughout the brooding and growing periods; (D,E) metabolites which were increased during brooding period but decreased during growing period; (F,G) metabolites which were decreased during brooding period but increased during growing period. Error bars represent the standard error values of relative abundance of metabolites among five liver samples.

Through Venn diagram analysis, we identified 34 metabolites that were co-altered in both brooding and growing periods (Fig. 6A). Among these 34 metabolites, four metabolites were continually increased (Fig. 6B) and six metabolites were continually decreased (Fig. 6C) throughout the brooding and growing periods. Meanwhile, 11 metabolites were increased during brooding period but decreased during growing period (Fig. 6D,E). However, another 13 metabolites were decreased during brooding period but increased during growing period (Fig. 6F,G). According to these analyses, we found that those four continually increased metabolites were related to the valine, leucine and isoleucine biosynthesis pathway and the pentose and glucuronate interconversions pathway, and those six continually decreased metabolites were mostly involved in the fatty acid metabolism and the steroid biosynthesis pathway. Meanwhile, those 11 metabolites, which were increased during brooding period but decreased during growing period, were mostly involved in some amino acid metabolic process, including the metabolism of the tryptophan, taurine, hypotaurine, glycine, serine, threonine, glutathione, arginine, and proline. Specifically, the lactic acid was also detected in the differentially expressed metabolites, which were increased during brooding period but decreased during growing period, which indicated that the glycolysis or gluconeogenesis processes were enhanced during the brooding period but weakened during the growing period. Moreover, 13 other metabolites, which were decreased during brooding period but increased during growing period, were proven to take part in the TCA cycle and other glycometabolism processes.

Furthermore, based on the metabolic pathway analyses, we found that valine, leucine and isoleucine biosynthesis pathway, steroid biosynthesis pathway, arginine and proline metabolism pathway, as well as the alanine, aspartate and glutamate metabolism pathway were co-altered in both brooding and growing periods. Accordingly, we found that the mostly altered metabolic processes were related to the amino acid metabolism process, which indicated that these amino acid played important roles in regulating the growth and development of the breeder roosters. By integrating the tendency of these distinguishing metabolites with the metabolic functional pathway changes from two different periods, we found that the amino acid metabolic process was continuously enhanced. However, the glucose and lipid metabolic functions of liver were initially decreased at brooding stage and then increased in growing period.

In the previous study, Tzur et al. (2009) found that the differentially expressed genes in the livers of mice from different periods were classified as related mainly to metabolic functions, which also proved that the metabolic function changed during the postnatal liver developmental process. Of these metabolic pathways, metabolic function such as cholesterol biosynthesis was an important member among the significantly over-represented pathways in postnatal liver-enriched genes, which agrees with our study and others (Turley et al., 1995). Peng et al. (2014) also reported significant changes in several metabolic functions during the postnatal liver development process, including metabolism of monocarboxylic acids, fatty acids, cellular ketones, lipids, and thioesters, which have also been proved in the present study. However, chickens are important oviparous animals whose main nutrients are obtained from the vitellicle during the embryonic period and the first 3 days after birth (Noy et al., 1996), thus, the glycometabolism, lipid and sterol metabolism in the newborn chicks are more enhanced than in the adult chickens.

Conclusively, in our study, we respectively identified 84 and 58 different kinds of distinguishing metabolites and further identified significantly changed metabolic pathways during the brooding and growing periods. The results obtained in this study suggested that there were extensively remodeled metabolic functions during the postnatal liver maturation process, and subsequently inspired us to provide suitable nutrients with the consideration of the metabolic changes during the different developmental periods. In addition, we specifically consider the differences in lipid and glucose metabolism between brooding and growing periods, and the same tendency of enhanced amino acid metabolic process with regards to these two developmental periods.

## MATERIALS AND METHODS

### Animals and sample collections

All experimental protocols and animals' managements in this study were approved by the Institutional Animal Care and Use Committee of the Northwest A&F University (Yangling, Shaanxi, China). Twenty 1-day-old healthy male Arbor Acres chicks, five 42-day-old healthy Arbor Acres breeder roosters, as well as five adult healthy Arbor Acres breeder roosters (35 weeks of age) were collected from Experimental Teaching Center of Animal Science of the Northwest A&F University (Yangling, Shaanxi, China). These randomly selected chickens were killed and immediately dissected. The left side livers were collected into Eppendorf tubes, and immediately frozen in liquid nitrogen. Then, all liver samples were stored at −80°C until being analyzed.

### Sample preparation for metabolomics research

Four liver samples of 1-day-old breeder roosters were mixed together as a pooled liver sample according to the weight of the livers. Then, five pooled liver samples from 1-day-old male chicks, five directly used liver samples from 42-day-old breeder roosters, and another five directly used liver samples from 35-week-old breeder roosters were used for GC-MS analyses.

Approximately 50 mg of liver sample were homogenized in 800 μl chloroform/methanol/water solvent (2:5:2) and the supernatant was collected. Then 640 μl of ice-cold methanol was added to the residue for another repeated extraction, after which the supernatants from the two extractions were pooled. Then 100 μl of the mixed solution were added to a glass vial with 10 μl of internal standards (0.05 mg/ml of 13C6-L-leucine and 13C6-15N L-isoleucine) and afterwards the mixture was dried under gentle nitrogen stream. The dried residuary sample was added to 30 μl of 20 mg/ml methoxyamine hydrochloride in pyridine, vortex-mixed for 30 s and incubated at 37°C for 90 min. After the incubation, 30 μl of N,O-bis(trimethylsilyl) trifluroacetamide with 1% trimethylchlorosilane were added into the mixture and incubated at 70°C for 60 min.

### GC-MS analysis

The derivative samples were analyzed using an Agilent 7890A gas chromatography system coupled to an Agilent 5975C Mass-Spring-Damper system (Agilent Technologies, CA, USA). Specifically, a HP-5 ms fused-silica capillary column (30 m×0.25 mm×0.25 μm; Agilent J&W Scientific, Folsom, CA, USA) was used to separate the derivatives. Helium (>99.999%) was used as a carrier gas at a constant flow rate of 1 ml/min through the column. Injection volume was 1 μl in splitless mode, and the solvent delay time was set at 6 min. The oven temperature was firstly held at 70°C for 2 min and then ramped to 160°C at a rate of 6°C/min, further increased to 240°C at the rate of 10°C/min and finally to 300°C at a rate of 20°C/min and held for 6 min. The temperatures of injector, transfer line, and electron impact ion source were set at 250°C, 260°C, and 230°C, respectively. The impact energy was set at 70 eV. Mass data was acquired in a full scan mode (m/z 50-600).

### Data preprocessing and statistical analysis

The acquired GC-MS data were processed as described in the previous study (Hu et al., 2017). For statistical analysis, the normalized data were imported to SIMCA P^+^ software (version 13.0, Umetrics, AB, Umea, Sweden). The model quality was described by the R^2^X or R^2^Y and Q^2^ values of principal component analysis (PCA) and partial least squares discriminant analysis (PLS-DA) (Hu et al., 2017). In order to avoid model over-fitting, a default 7-round cross validation in SIMCA P^+^ software was performed throughout so that the optimal number of principal components could be determined. The variable importance in projection (VIP) values of the orthogonal projection of latent structures–discriminant analysis (OPLS-DA) model greater than 1 and *P* values of univariate statistical analysis less than 0.05 were identified as significantly distinguishing metabolites. Fold change was calculated as binary logarithm of average normalized peak intensity ratio between the compared groups. Then, the AMDIS software (Agilent Technologies, CA, USA) was applied to deconvolute mass spectra from raw GC-MS data. The pathway analyses based on distinguishing metabolites were performed by MetaboAnalyst 3.0 (http://www.metaboanalyst.ca) (Xia et al., 2015).
